# Role of auxin during intercellular infection of *Discaria trinervis* by *Frankia*

**DOI:** 10.3389/fpls.2014.00399

**Published:** 2014-08-21

**Authors:** Leandro Imanishi, Francine M. Perrine-Walker, Adama Ndour, Alice Vayssières, Genevieve Conejero, Mikaël Lucas, Antony Champion, Laurent Laplaze, Luis Wall, Sergio Svistoonoff

**Affiliations:** ^1^Laboratorio de Bioquímica Microbiología e Interacciones Biológicas en el Suelo, Departamento de Ciencia y Tecnología, Universidad Nacional de QuilmesBernal, Argentina; ^2^Groupe Rhizogenèse, Institut de Recherche pour le Développement, UMR DIADEMontpellier, France; ^3^LAPSE and Laboratoire Commun de Microbiologie IRD/ISRA/UCAD, Centre de Recherche de Bel-AirDakar, Senegal; ^4^Institut National de la Recherche Agronomique, Plateforme PHIV, CiradMontpellier, France

**Keywords:** auxin, actinorrhiza, systems biology, endosymbiosis, model, AUX1, PIN, nitrogen fixation

## Abstract

Nitrogen-fixing nodules induced by *Frankia* in the actinorhizal plant *Discaria trinervis* result from a primitive intercellular root invasion pathway that does not involve root hair deformation and infection threads. Here, we analyzed the role of auxin in this intercellular infection pathway at the molecular level and compared it with our previous work in the intracellular infected actinorhizal plant *Casuarina glauca*. Immunolocalisation experiments showed that auxin accumulated in *Frankia*-infected cells in both systems. We then characterized the expression of auxin transporters in *D. trinervis* nodules. No activation of the heterologous *CgAUX1* promoter was detected in infected cells in *D. trinervis*. These results were confirmed with the endogenous *D. trinervis* gene, *DtAUX1*. However, *DtAUX1* was expressed in the nodule meristem. Consistently, transgenic *D. trinervis* plants containing the auxin response marker *DR5:VENUS* showed expression of the reporter gene in the meristem. Immunolocalisation experiments using an antibody against the auxin efflux carrier PIN1, revealed the presence of this transporter in the plasma membrane of infected cells. Finally, we used *in silico* cellular models to analyse auxin fluxes in *D. trinervis* nodules. Our results point to the existence of divergent roles of auxin in intercellularly- and intracellularly-infected actinorhizal plants, an ancestral infection pathways leading to root nodule symbioses.

## Introduction

Actinorhizal symbiosis comprise more than 200 species from 8 families distributed within the orders Fagales, Cucurbitales and Rosales, which form nitrogen fixing nodules with the soil actinomycete *Frankia*. Unlike legumes whose nodules are characterized by a peripheral vascular system, actinorhizal nodules present a central vascular bundle that structurally resembles a lateral root. Moreover, the actinorhizal nodule develops from the pericycle, like lateral roots (Wall, 2000; Svistoonoff et al., 2014).

Auxins are involved in many developmental processes and play a major role in lateral root initiation, differentiation and maintenance (Lavenus et al., 2013). Several studies revealed that auxins are also involved in nodule development in legumes (Mathesius et al., 1998; De Billy et al., 2001; Pacios-Bras et al., 2003). It has been suggested that rhizobia inhibit auxin transport at the early stages of the symbiotic interaction in legumes forming indeterminate type of nodules (Mathesius et al., 1998; De Billy et al., 2001), but not in *L. japonicus* that forms determinate type of nodules (Pacios-Bras et al., 2003). In both cases an accumulation of auxin occurs at the site of nodule initiation and auxin is supposed to stimulate cell divisions in the cortex and pericycle that lead to the formation of nodule primordia (Roudier et al., 2003). Recently we demonstrated that auxin also plays an important role during actinorhizal nodule development in *C. glauca*. We showed that inhibition of auxin influx transport by the competitive inhibitor naphthoxyacetic acid (1-NOA) perturbs nodule formation (Péret et al., 2007). Auxins were detected in *Frankia* cultures and in *Frankia*-infected cells in *C. glauca* nodules (Perrine-Walker et al., 2010). Those infected cells were shown to express an auxin influx carrier (*CgAUX1*) whereas a PIN1-like auxin efflux carrier was detected in surrounding uninfected cells (Perrine-Walker et al., 2010). Using computer simulations we showed that the pattern of transporter distribution leads to auxin accumulation in infected cells, where auxins possibly induce changes in gene expression, cell metabolism, or in the cell wall properties (Perrine-Walker et al., 2010, 2011).

*Discaria trinervis*, an actinorhizal shrub belonging to the *Rhamnaceae* family, was recently chosen as a model plant for the study of the intercellular infection pathway in actinorhizal symbiosis (Imanishi et al., 2011). Whereas the molecular basis of nodulation in plants infected through root hairs has been extensively studied in Legumes and in actinorhizal plants, little is known about plants infected through the intercellular infection pathway which is found in about 75% of actinorhizal genera and is possibly an ancestral process which led to the more sophisticated root hair infection (Svistoonoff et al., 2014). The intercellular infection pathway in *D. trinervis* begins with the invasion of the root epidermal and cortical cells by *Frankia* that penetrates the root tissue through the intercellular spaces in between adjacent epidermal root cells. *Frankia* infection triggers cell divisions in the pericycle leading to the formation of a nodule primordium. The nodule primordium gives rise to the mature nodule comprising several lobes. Each lobe is structurally very similar to a lateral root with a central vascular bundle, a well-developed cortex and an apical meristem. Neither root hair deformation nor infection thread formation takes place as in *C. glauca*. The prenodule, a small protuberance that originates from cortical cells divisions beneath of a *Frankia* infected root hair, is also absent in the intercellular infection in *D. trinervis*. In contrast to *C. glauca*, *Frankia* remains intercellular during the early steps of the infection process in *D. trinervis* and only becomes intracellular once the bacteria reach the cortical cells of nodule primordia (Valverde and Wall, 1999). Very little is known about the mechanisms involved in intercellular infection in actinorhizal plants. Here, we analyzed the role of auxin in intercellular infection of *D. trinervis* by *Frankia*.

## Materials and methods

### Plant material, bacterial strains and growth conditions

Seeds of *D. trinervis* were collected in Pampa de Huenuleo (Bariloche, Argentina). Seeds were surface sterilized as previously described (Valverde and Wall, 1999). Germination was performed in Petri dishes containing distilled water solidified with 1% agar. Two weeks after germination, seedlings were transferred to pots containing BD medium as described (Svistoonoff et al., 2010). For the auxin-influx carrier inhibition experiments, seedlings were transferred to pouches (Mega International, Minneapolis, MN, USA) containing BD medium. 25 μ M of 1-naphtoxyacetic acid (1-NOA) were added to the growth medium 2 weeks before or at the time of inoculation and solutions with or without 1-NOA were renewed every week; 34–55 plants were analyzed for each condition. Statistical analysis was performed using One-Way ANOVA and the Tukey-Kramer multiple comparison test implemented in Rcommander. The *A. rhizogenes* ARqua1 (Boisson-Dernier et al., 2001) strain was grown at 28°C as described (Imanishi et al., 2011). The *Frankia* BCU110501 (Chaia, 1998) strain was cultivated at 28°C in a modified BAP medium supplemented with glucose as a carbon source (Murry et al., 1984).

### *DtAUX1* identification sequence analysis and quantitative PCR

To identify *AUX1* homologs in *D. trinervis* we used the set of degenerate primers used to isolate *CgAUX1* and *CgLAX3* in *C. glauca* (Péret et al., 2007). cDNA and Genomic DNA from *D. trinervis* were isolated as described for *C. glauca* (Péret et al., 2007). The full-length DNA sequence of *DtAUX1* gene was obtained using the Universal Genome Walker kit (CLONTECH) using the primers DtAux1_GSP1_5′ 5′-CTGATAAGATAAGCCGTCCAGCTTCCGA-3′, DtAux1_GSP2_5′ 5′-ATGATGCCGGAAAGCAAGCCCAATTGAG-3′, DtAux1_GSP1c_5′ 5′-CTTCCTCTGCTTGTTTCTGAGCCAACAT-3′, DtAux1_GSP2c_5′ 5′-ACGCAGCCCCAGAAAACGAAAGCCAATA-3′, DtAux1_GSP1_3′ 5′-TCGATGACCGTTTGGATAAGAGAACTTG-3′, DtAux1_GSP2_3′ 5′-GGTCTTGGGATGACCACCTATACGGCTT-3′, DtAux1_GSP1c_3′ 5′-TTTGTGGTAGGGTTTGGGTTCGGTGGAT-3′ and DtAux1_GSP2c_3′ 5′-ATACCGGCACCTCCGCATCACTAGAAAA-3′. CDS sequence was amplified by PCR on cDNA extracted from roots using primers DtAux1_cDNA_Fw 5′-ATGTTGGCTCAGAAACAAGCAG-3′, and DtAux1_cDNA_Rv 5′-CTAGTGATGCGGAGGTGCC-3′. Quantitative PCR was performed on cDNAs extracted from nodules and non-inoculated roots as described (Svistoonoff et al., 2013) using primers DtAux1F 5′-ACGGCATGACCACCAAAGG and DtAux1R 5′GGTTACTCACTCTGCTCCATCC-3′ to amplify a DtAux1 fragment and DtUbiF 5′-TACCACCACGAAGACGGAGGAC-3′ and DtUbiR 5′-GGAAGCAGTTGGAGGATGGAAGG to amplify an ubiquitin fragment used as an internal standard. The sequence of *DtAUX1* was deposited at Genbank and was given the accession number KM200713.

For the phylogenetic analysis coding sequences of the AUX-LAX family of auxin influx carriers were retrieved by family blast search in the Phytozome v9.1 database (www.phytozome.org) using the coding sequence of *DtAUX1* as the query. Sequences of AUX-LAX family from *D. glomerata* were retrieved using BLAST search from *D. glomerata* nodule transcriptome database (Demina et al., 2013; https://fido.nsc.liu.se/datisca/nodule/) and the coding sequences of all AUX-LAX family from *A. thaliana* as the query. The phylogenetic tree was constructed using the coding sequences as described (Svistoonoff et al., 2013) except that RAxML7.6.6 (Stamatakis, 2006) was used to calculate the phylogenetic tree with default parameters including automatic halt of bootstrap.

### Generation of *ProDTAUX1:gus* construct

A 1593 bp genomic DNA fragment upstream of the *DtAUX1* start codon was amplified by PCR with Phusion High Fidelity Polymerase (NEB) and cloned into pDISC (Fliegmann et al., 2013) binary vector by Golden Gate cloning method (Engler et al., 2008) together with a GUS reporter gene. The promoter region of the DtAUX1 gene was amplified using the primers ProDtAUX1_FW3 5′-CTCGGTCTCGaaatGAAATTAATTGGGAAATTAAATTCATGGAATTATTG-3′ and ProDtAUX1_R 5′-CTCGGTCTCGcattGTTTATATCTTGGTAGATCTGAAACATATA-3′ and *D. trinervis* DNA as template. The CDS of GUS reporter gene was amplified with the primers Cg12_GUS_Fw 5′-CTCGGTCTCGaatgTTACGTCCTGTAGAAACCC-3′ and Cg12_GUS_Rv 5′-CTCGGTCTCGcgtaCCCGATCTAGTAACATAGATGAC-3′ using the pBI101.3 binary vector (CLONTECH) as template. The primers incorporate a BsaI restriction site (underlined) at the end of both amplification products that allows a correct assembly of the *DtAUX1* promoter sequence to the *GUS* reporter gene in the pDISC binary vector. Both amplification products were first cloned with CloneJET PCR cloning kit (Thermo Scientific) and sequenced to confirm the absence of any PCR induced mutation. For the Golden Gate reaction, each plasmid was quantified and added to the reaction mix in an equal molar ratio of 50 fmoles each. The reaction mix was completed with 15 units of high concentration HC T4 DNA ligase (Promega), 2.5 units of BsaI (NEB), 10 × T4 DNA ligase buffer (Promega) and water up to 15 μ L. The restriction-ligation reaction was performed in a thermocycler with the following program: 50 cycles of 2 min incubation at 37°C and 5 min at 16°C, followed by 5 min incubation at 50°C and then a heat inactivation at 80°C for another 5 min. 5 μ l of the reaction were used to transformed *E. coli* TOP10 chemically competent cells by heat shock.

### Composite plants generation

The pDISC-ProDtAUX1:GUS and the DR5:Venus binary vectors were introduced into *A. rhizogenes* ARqua1 strain by electroporation. Transformation of *D. trinervis* was performed using the *ex vitro* method described in Imanishi et al. (2011). Transgenic roots were identified by detecting DsRED or Venus fluorescence using a MZFLII stereomicroscope (Leica) with GFP2 or G filter sets or using a blue LED bulb (Orbitec) and 2846 protection glasses with orange lens (3M).

### Histochemical gus assays and microscopy

GUS assays were performed as described (Imanishi et al., 2011). Sections were prepared and stained as described (Imanishi et al., 2011; Svistoonoff et al., 2013). Microscopy observations were performed with a DMRB (Leica) and an AX10 (Zeiss) microscope and images were captured using an MP5 (Qimaging) digital camera.

### PAA and PIN1 immunolocalization

For PAA and PIN1 immunolocalization assay, fresh nodules section of 50 to 60 μm were obtained with a HM650V vibratome (MicroM), they were fixed and incubated with the corresponding antibody as previously described (Perrine-Walker et al., 2010). Sections were then mounted in Mowiol (Calbiochem) and visualized on a Zeiss confocal microscope 510 META.

## Results

### Actinorhizal nodule formation is inhibited by the auxin influx inhibitor 1-NOA

**Figure 1:**
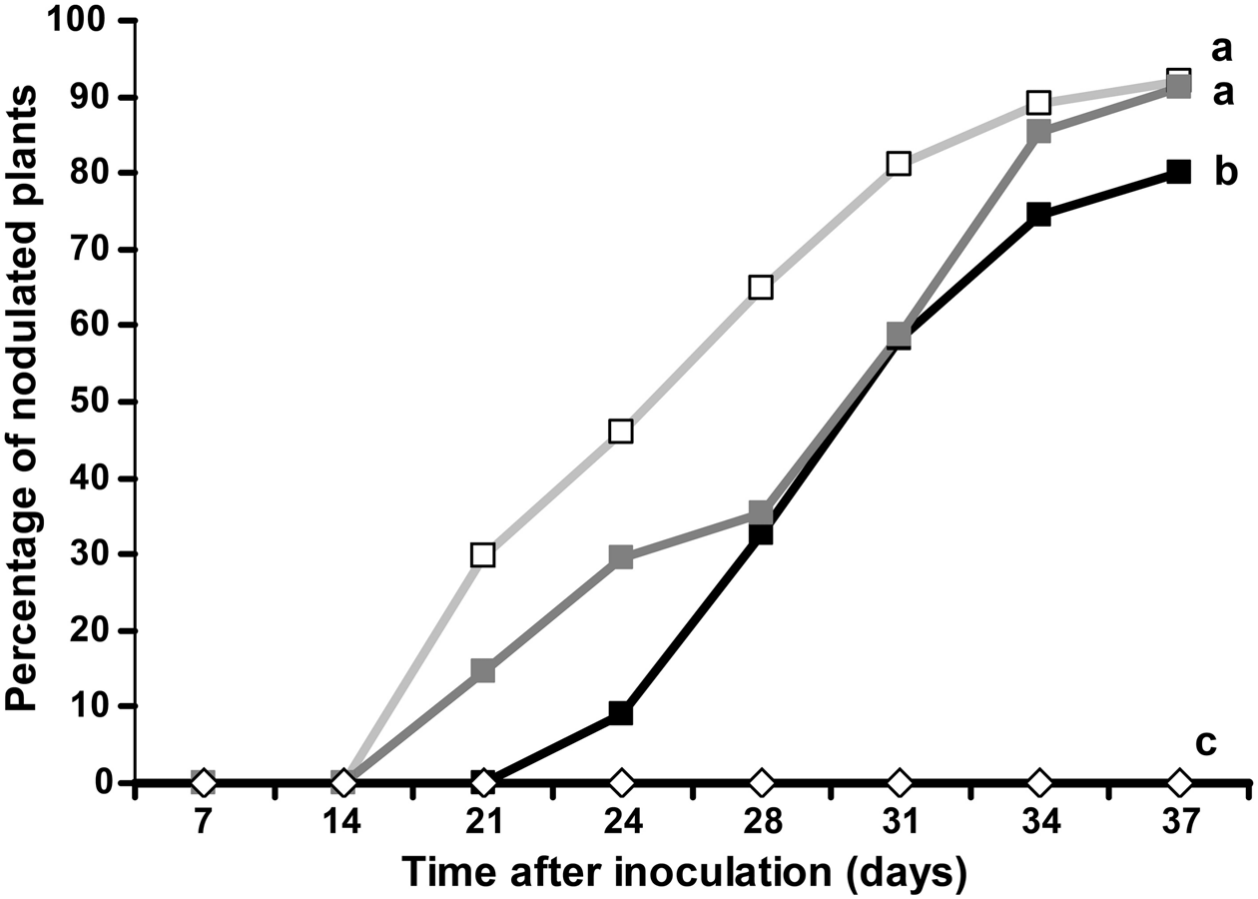
**Auxin influx transport inhibitor 1-NOA negatively affects nodulation in *D. trinervis***. Percentage of plants showing nodules after inoculation with *Frankia* in the presence of 1-NOA from before inoculation (black line/solid box), from the moment of inoculation (dark gray line/solid squares) or without inhibitor (gray line/empty squares). Nodulation was not observed in non-inoculated control plants (empty diamonds). Letters indicate statistically different groups based on the Tukey-Kramer multiple comparison procedure (*P* < 0.01).

1-naphtoxyacetic acid (1-NOA), a competitive inhibitor of auxin influx is known to inhibit auxin influx transport in *A. thaliana* and to perturb actinorhizal nodule formation in *C. glauca* (Péret et al., 2007). Similarly, we investigated the effect of 1-NOA on nodulation of *D. trinervis*. Plants grown in hydroponics were inoculated with *Frankia* and 25 μ M 1-NOA was added either 2 weeks before the inoculation with *Frankia* or from the moment of inoculation. Plants watered with 1-NOA showed a 10 d delay in nodulation compared to untreated controls (Figure 1, black vs. light gray lines). The percentage of nodulated plants was lower for plants treated with 1-NOA before inoculation while plants for which the 1-NOA treatment started at the time of inoculation showed an intermediate behavior (Figure 1, dark gray line), reaching values close to the controls by the end of the experiment. We were unable to detect any difference of plant dry weight between treatments suggesting that effect of 1-NOA cannot be attributed to reduced plant growth. Altogether, these results indicate that inhibition of auxin influx activity perturbs nodule formation in *D. trinervis* similarly to what was observed in *C. glauca*.

### Distribution of auxin accumulation and auxin perception in *D. trinervis*

**Figure 2:**
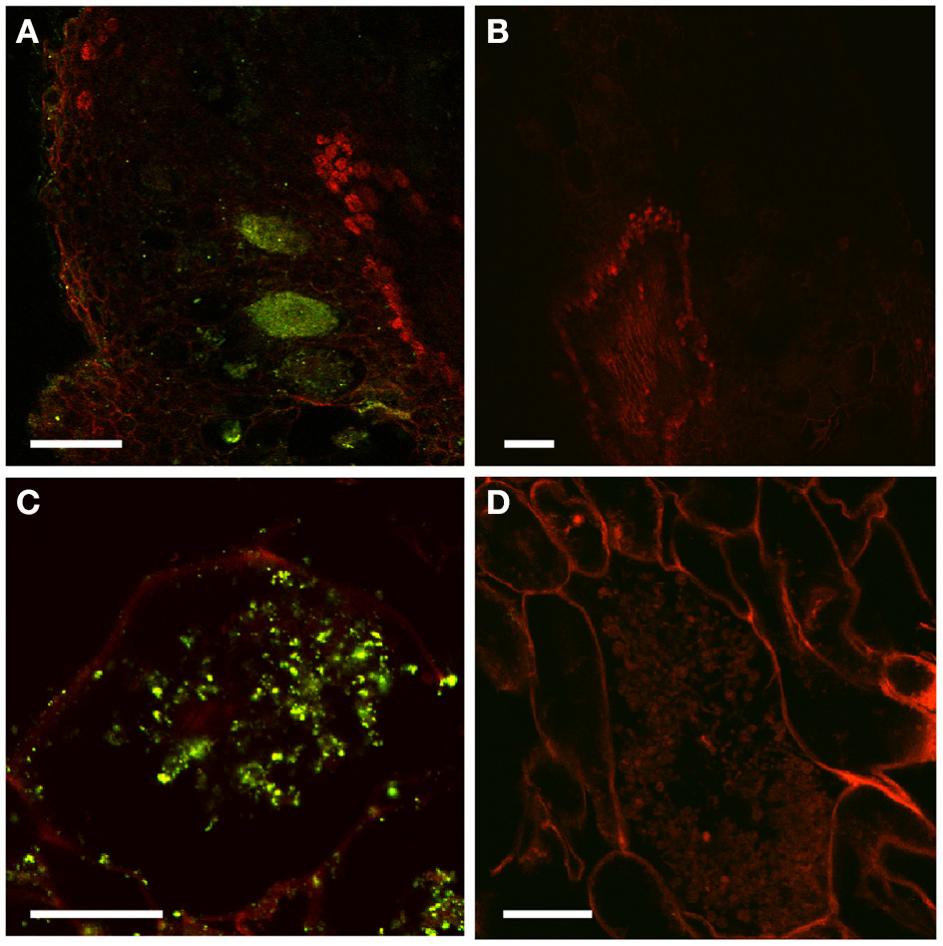
**Immunolocalization of PAA in *D. trinervis* nodules. (A–C)**. A strong signal is detected in cells infected by *Frankia*. No signal is present in the vascular bundle or in non-infected cells. **(B–D)** No signal is detected in control sections incubated with the secondary antibody alone. Scale bars: A, B: = 50 μm; C, D: 25 μm.

To further study the role of auxin in *D. trinervis*, we analyzed the distribution of auxins and the sites of auxin perception. First we looked at the distribution of the auxin phenylacetic acid (PAA) in sections of mature nodules incubated with a polyclonal anti-PAA antibody. High levels of PAA were detected within the characteristic hypertrophied cortical nodule cells infected by *Frankia* while no signal was detectable in the smaller uninfected cells (Figures 2A,C). No fluorescence was detected in control sections incubated with the secondary antibody alone (Figures 2B,D). Hence, the auxin PAA accumulates in *Frankia*-infected cells in *D. trinervis* nodules.

**Figure 3:**
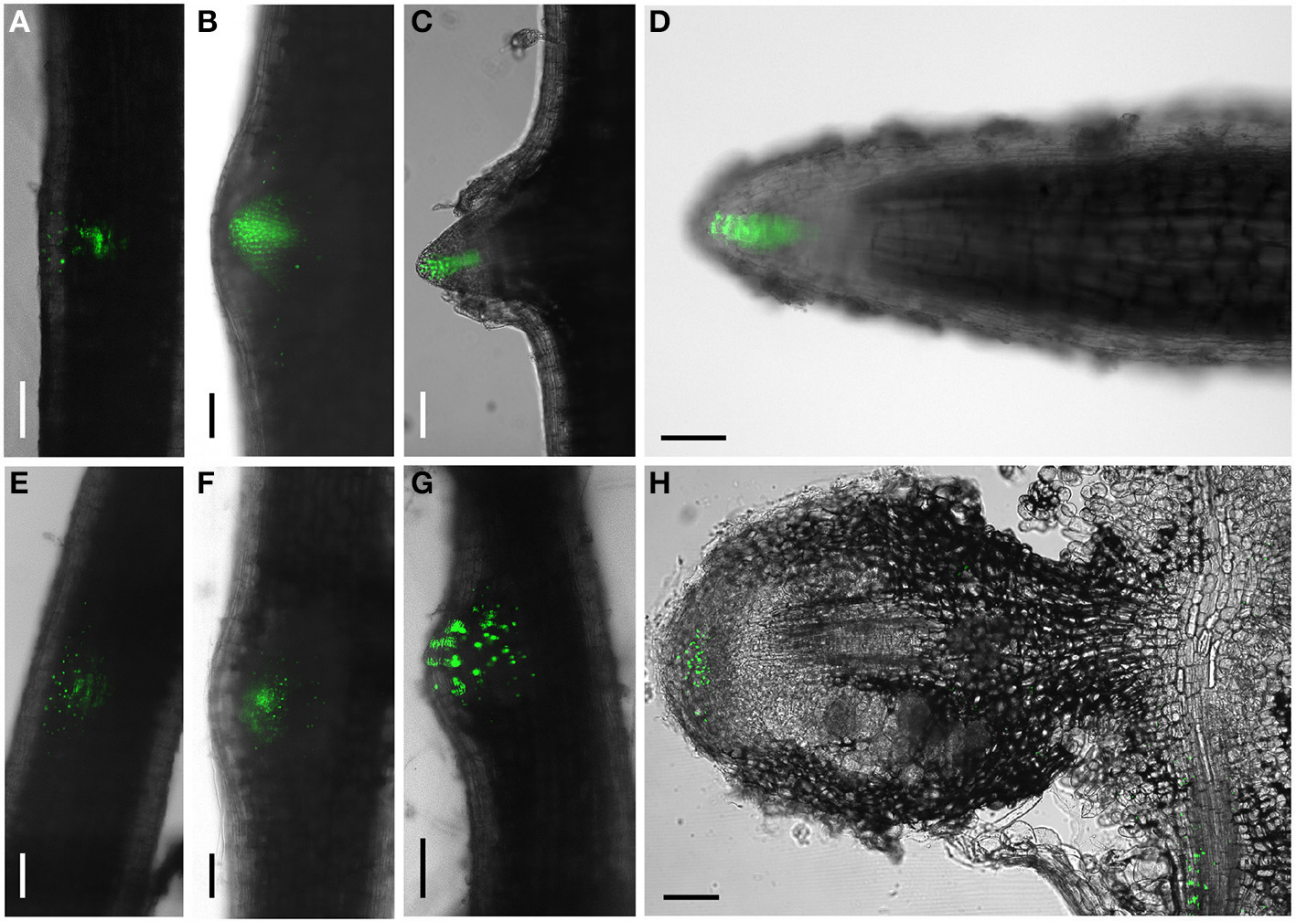
***DR5:VENUS:NLS* expression pattern during lateral root and nodule development. (A–C)** Lateral root: DR5 activation is gradually concentrated at the tip of developing lateral roots. **(D)** a mature lateral root showing VENUS fluorescence concentrated in the columella. **(E–H)** Nodule primordia; no concentration gradient of VENUS expression is observed in the tip of the emerging nodule primordium. **(G)** Longitudinal section of a mature nodule showing DR5 activation in the meristematic region. Scale bars:100 μm.

In a second series of experiments we generated composite transgenic *D. trinervis* plants expressing a *DR5:Venus:NLS* construct (Heisler et al., 2005). DR5 is widely used as a molecular marker of auxin perception (Ulmasov et al., 1997; Benková et al., 2003) and the *DR5:Venus:NLS* fusion is particularly sensitive as the fluorescence is concentrated in nuclei. Before the inoculation with *Frankia*, strong DR5 activity was detected showing a continuous gradient of expression in the root tips, mostly at the columella root cap and also in lateral root primordia even before their emergence (Figures 3A–D). Upon inoculation with *Frankia*, plants started to form nodule primordia at 5 dpi. Nodules began to emerge at 9 dpi and mature nodules were observed at 21 dpi. VENUS fluorescence was detected starting from 5 dpi in nodule primordia (Figures 3E–G). In mature nodules, activation of DR5 was restricted to the meristematic zone (Figure 3H). The expression in meristems of nodules was generally more scattered compared to lateral root primordia. We conclude that DR5 is activated in similar tissues during the formation of nodules and lateral roots. Remarkably, although PAA was detected in infected cells, we were unable to detect any activation of DR5 in those cells (Figure 3H). To investigate the responsiveness of DR5 to auxin, roots were incubated with 10 μ M NAA. 24 h after the addition of auxin, DR5 activation was observed in an extended zone at the root tip but also in a zone situated few mm shootwards (Figure S1 in Supplementary Material). Similar results were described for DR5 in Arabidopsis (Ottenschläger et al., 2003) indicating that DR5 regulation is well conserved between both species. This indicates that auxin perception occurs in *D. trinervis* nodule meristem. The absence of DR5 activity in *Frankia*-infected cells might be due to a specific cellular context that prevents this marker from functioning.

### Distribution of auxin carriers in *D. trinervis*

**Figure 4:**
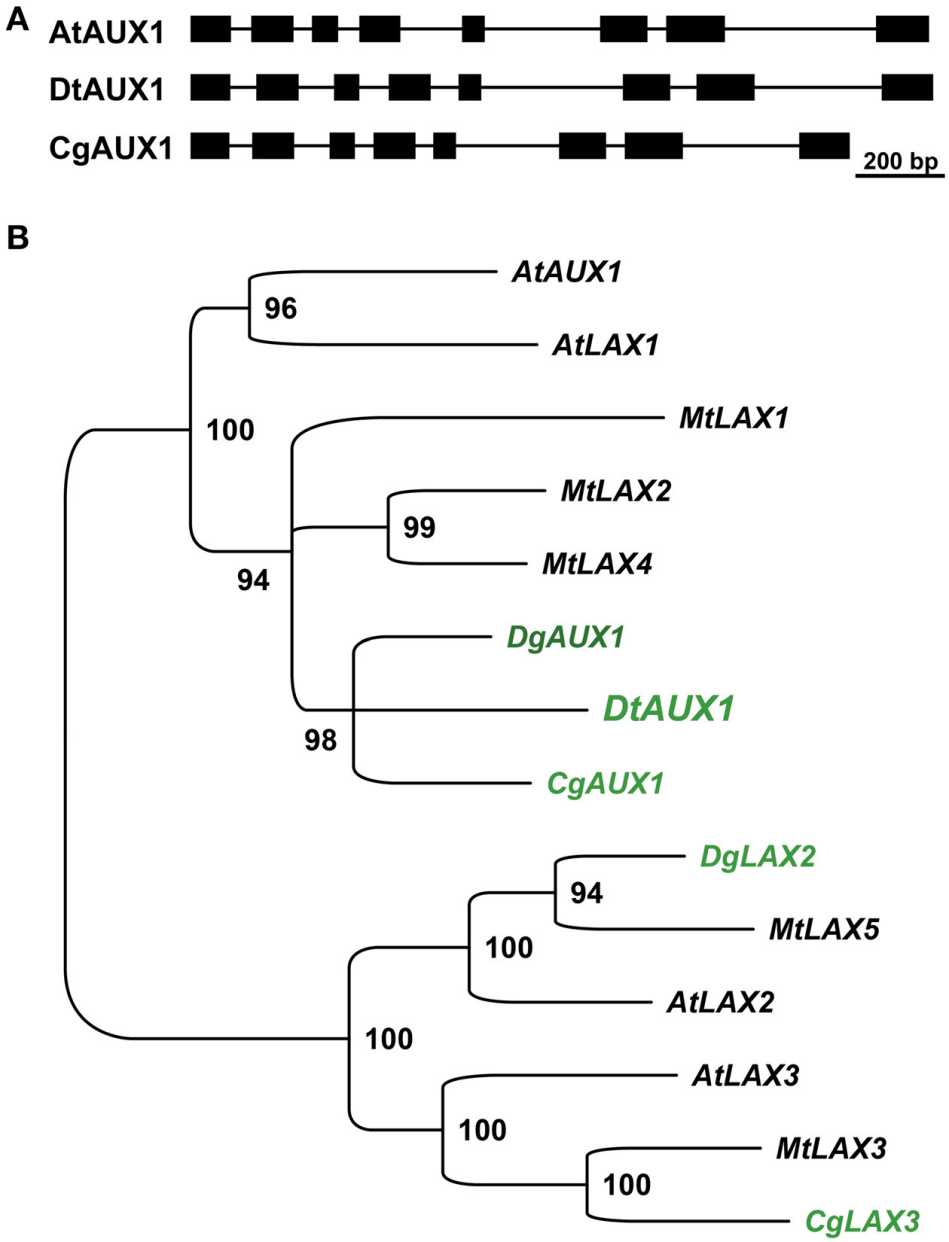
***DtAux1* encodes a putative orthologue of *CgAUX1*/*AtAUX1***. **(A)** Exon-intron structure of *DtAUX1* compared to *AtAUX1* and *CgAUX1*. Exons are displayed as black boxes. **(B)** Maximum likelihood phylogeny obtained with the coding sequences of *AUX*-*LAX* genes. DtAUX1 clusters together with CgAUX1 and DgAUX1 and belongs to the AUX/LAX subclass of auxin influx carriers. Numbers indicate the percentage of bootstrap support.

**Figure 5:**
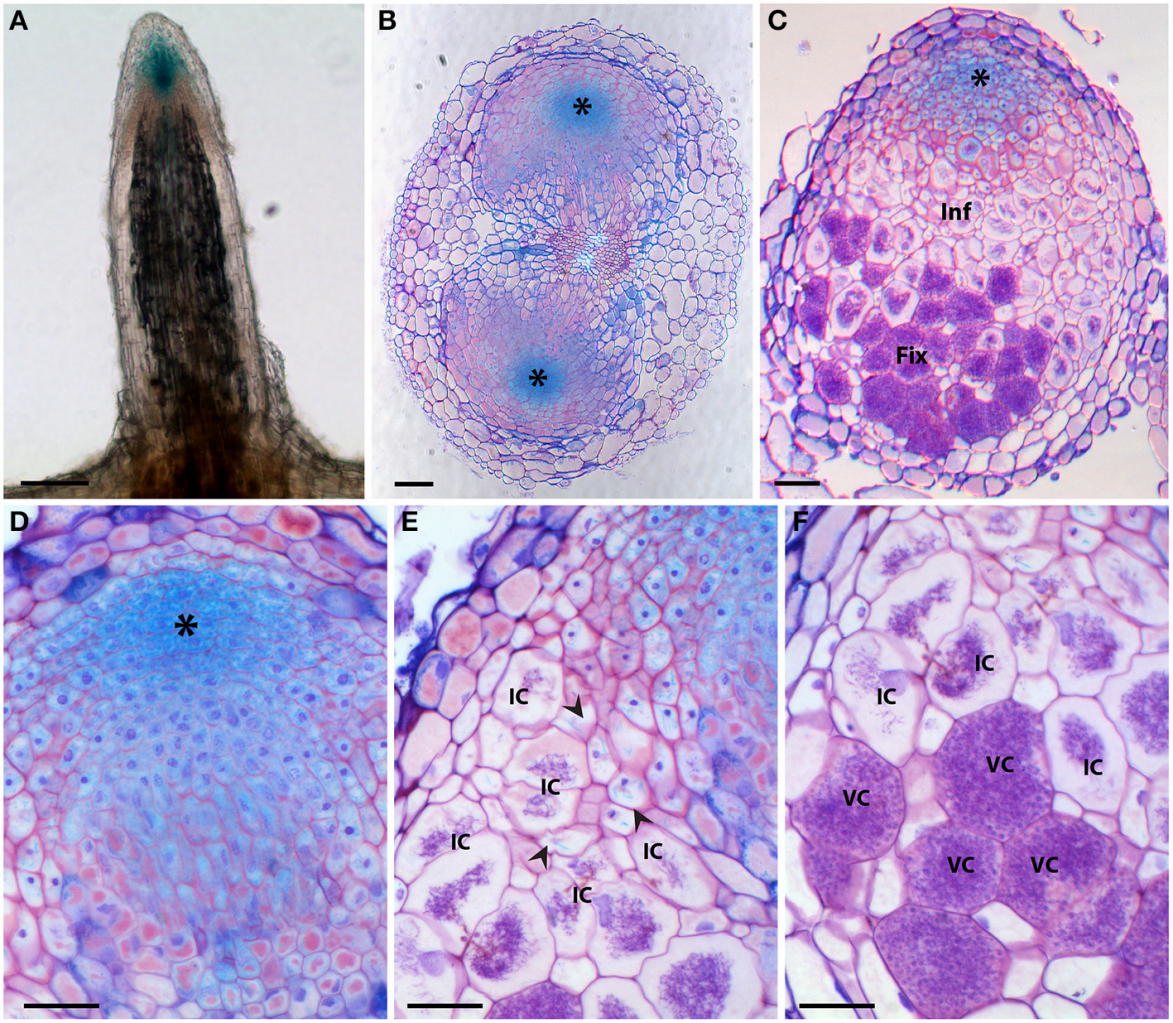
**Histochemical localization of β-glucuronidase (GUS) activity in *D. trinervis* roots expressing a *ProDtAUX1:GUS* construct**. **(A)** Non-inoculated lateral root; blue staining is detected in the root tip. **(B)** Cross section of an inoculated root 5 days after inoculation (dai) showing two nodule primordia growing from the pericycle at opposite xylem poles. *DtAUX1* expression is observed in the meristematic cells (asterisk). **(C)** Longitudinal section of a fully developed nodule 21 dai. Cells containing *Frankia* hyphae are stained in purple. GUS activity is intense in the meristematic region, still visible in the infection zone (Inf), in non-infected cells, and non-detectable in the fixation zone (Fix). **(D–E)** Magnified images of **(C)**. **(D)** Meristematic zone. **(E)** Infection zone: *DtAUX1* expression is limited to non-infected cortical cells (arrowheads) surrounding the highly hypertrophied infected cells (IC). **(F)** Fixation zone: no *DtAUX1* activation is detected in the hypertrophic cells filled with *Frankia* that have already differentiated nitrogen-fixing vesicles (VC). Sections **(B–F)** were stained with toluidine blue. Scale bars: 100 μm **(A–C)**, 50 μm **(D–F)**.

The perturbation of *D. trinervis* nodulation caused by 1-NOA prompted us to investigate the endogenous genes of *D. trinervis* related to auxin influx carriers. Using the degenerated primers used for the identification of *CgAUX1* and *CgLAX3* in *C. glauca* we obtained a single PCR product for *D. trinervis*. Starting from this sequence we identified the complete Coding DNA Sequence (CDS) and the genomic sequence including a 1778 bp upstream the ATG. We called this gene *DtAUX1*. *DtAUX1* contains a CDS encoding a 1446 aa protein which shows 89 and 83% sequence identity to *C. glauca* CgAUX1 and *A. thaliana* AtAUX1 proteins respectively. The genomic DNA sequence was 3312 bp long from start to stop codon and the exon-intron structure of the gene was conserved with respect to *CgAUX1* and *AtAUX1* (Figure 4A). A maximum likelihood phylogenetic analysis including DtAUX1 and members of the AUX-LAX family from *A. thaliana*, the legume *M. truncatula* and the actinorhizal plants *C. glauca* and *Datisca glomerata*, showed that DtAUX1 clusters in the same strongly supported group as CgAUX1 and DgAUX1 which belongs to the AUX1/LAX subfamily (Figure 4B). Using quantitative PCR we monitored *DtAUX1* expression and found twice as many transcripts in nodules compared to roots (data not shown). To further investigate the spatio-temporal pattern of expression of *DtAUX1*, we generated transgenic *D. trinervis* roots containing a transcriptional fusion between a promoter sequence 1593 bp upstream the ATG and the GUS reporter gene. A strong GUS activity was found in root tips of non-inoculated roots, particularly in the columella cells, the quiescent center and the stem cells (initials), and in lateral root primordia and the vascular tissues (Figure 5A). No GUS activity was detected in the epidermis or the cortex after inoculation with *Frankia*. However, strong activation of ProDtAUX1 was visible in nodule primordia starting from 5 dai (Figure 5B). In mature nodules, strong GUS activity was detected in the meristematic zone and the vasculature (Figures 5C–F). We were unable to detect any activation of ProDtAUX1 in infected cells (Figures 5E,F). We conclude that in *D. trinervis*, ProDtAUX1 is active in the apical meristem and the vascular tissues of both roots and nodules but not during the infection by *Frankia*. Similar results were obtained with the promoter from *C. glauca* (*Pro_CgAUX1_:GUS*; Figure S2 in Supplementary Material).

**Figure 6:**
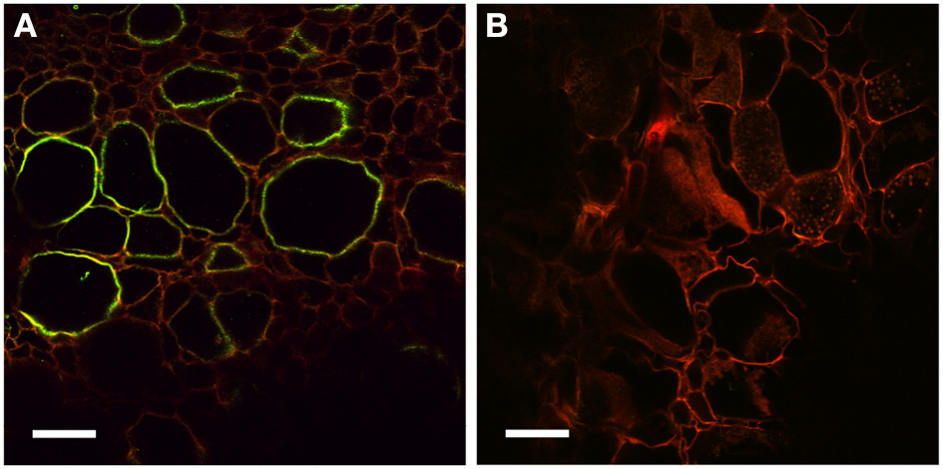
**Immunolocalization of PIN1-like proteins**. **(A)** Longitudinal section of a mature nodule incubated with Anti-PIN1 antibodies and FITC labeled secondary antibodies: a strong signal is detected in the plasma membrane of hypertrophied cortical cells infected by *Frankia*. **(B)** Control section incubated with the secondary antibody alone where no signal is detectable. Scale bars: 50 μm.

In addition to auxin influx carriers, we also analyzed the distribution of auxin efflux carriers using polyclonal anti-AtPIN1 antibodies that were used to determine the distribution of PIN1-like proteins in *C. glauca* nodules (Perrine-Walker et al., 2010). Sections of mature nodules showed a strong signal in the infection zone, in the membranes of hypertrophied cells infected by *Frankia* (Figure 6A). Remarkably, no signal could be detected in the small uninfected cells surrounding infected cells where PIN-1 was found in *C. glau*ca nodules (Perrine-Walker et al., 2010). Therefore, the distribution of auxin transporters in intercellularly-infected *D. trinervis* is different from the one observed during intracellular infection in *C. glauca*.

### Distribution of auxin carriers is insufficient to explain auxin accumulation in infected cells and meristem of *D. trinervis* nodules

**Figure 7:**
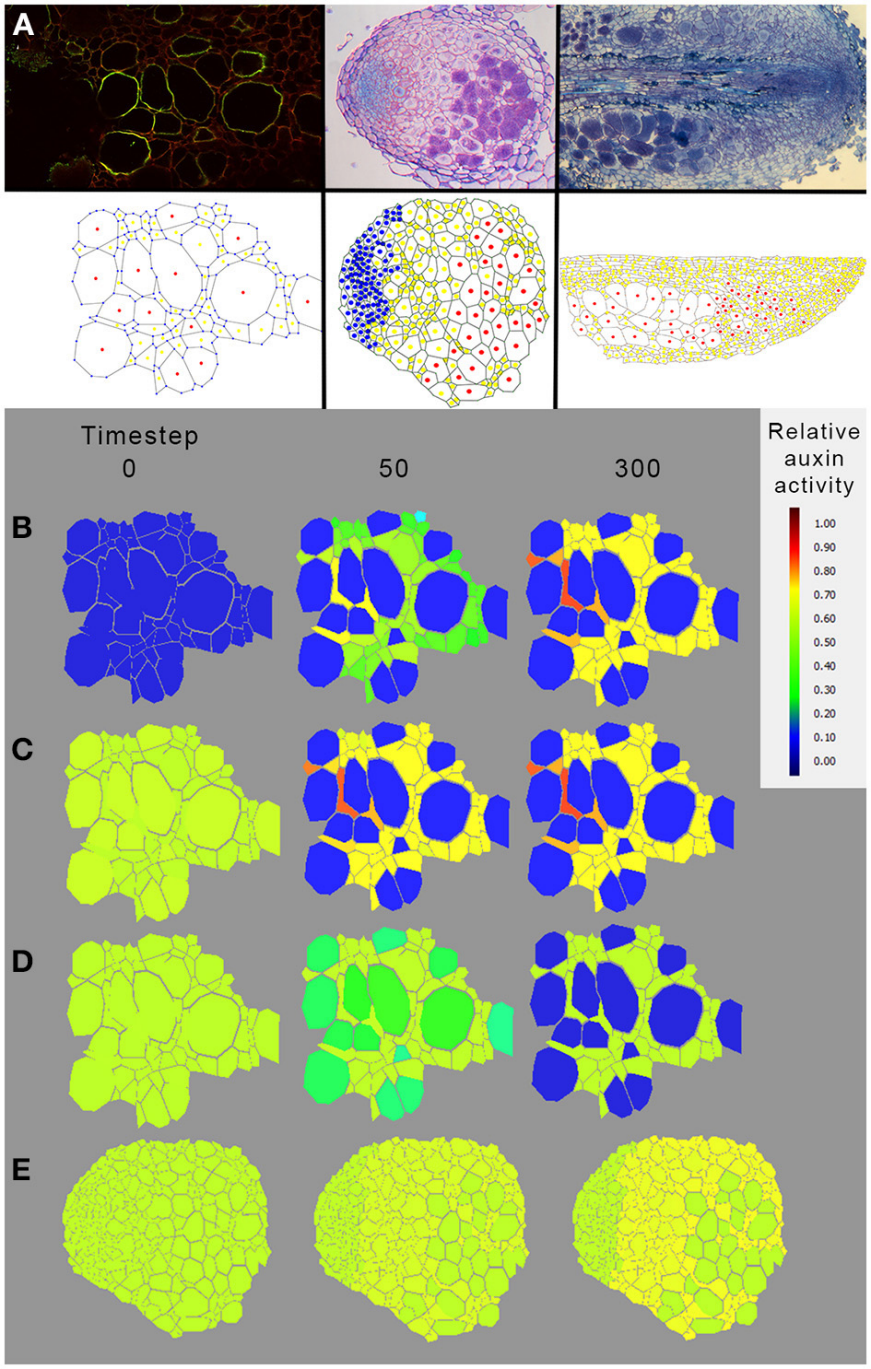
**Modeling auxin fluxes in *D. trinervis* nodules. (A)** Example of digitization of nodule tissue. Images of nodule tissue autofluorescence (top panel) were used as a base for manual digitization of the geometry in a virtual representation of the nodule (Perrine-Walker et al., 2010). Information regarding the infection of the cells was added in the virtual tissue (infected cells are denoted in red, uninfected cells in yellow and meristematic cells in blue). **(B–E)** Distribution of auxin in a virtual nodule with meristematic cells expressing DtAUX1 and infected cells expressing a PIN1-like auxin efflux carrier. Auxin flux simulations were conducted with either no initial auxin and basal auxin production throughout the tissue **(B**), basal initial auxin level throughout the tissue **(C)**, or an auxin source located within the *Frankia* compartment (to simulate auxin production by *Frankia*, **D**,**E**). In all cases, auxin was predicted to accumulate in non-infected cells of *D. trinervis* actinorhizal nodules rather than in infected and meristematic cells. Exploration of the model parameter space did not reveal a parameter configuration for which the biological auxin accumulation could be reproduced by the model based on DtAUX1 and DtPIN1 localization (data not shown). This suggests that the mechanisms leading to auxin accumulation in those cells do not rely exclusively on *D. trinervis* PIN1-like and DtAUX1.

The immunolocalization of PIN1 like proteins revealed that the auxin efflux carriers of *D. trinervis* are located at the membrane of infected cells in *D. trinervis* while auxin (PAA) is present in infected cells and the influx carriers *DtAUX1* is expressed in the nodule meristem (Figure 5). To test the impact of this localization on the pattern of auxin accumulation, we used an integrative biology approach similar to the one that was used to study auxin fluxes in *C. glauca* nodules (Perrine-Walker et al., 2010). Images of sections of *D. trinervis* nodules were digitized and used to generate virtual tissues integrating auxin physiology mechanisms (Figure 7A). Three *in silico* models of nodular tissues were generated from images of three different nodules. Up to three different cell types corresponding to infected, uninfected and meristematic cells were identified in the virtual tissues. DtPIN1 and DtAUX1 transport activities were respectively located to infected and meristematic cell membranes, in accordance with the experimental results (Figure 7A).

Auxin flux simulations were conducted with either (i) no initial auxin and basal auxin production throughout the tissue (Figure 7B), (ii) a basal initial auxin level throughout the tissue (Figure 7C), or (iii) an auxin source located within the *Frankia* compartment (to simulate auxin production by *Frankia*, Figure 7E). In all cases, auxin was predicted to accumulate in non-infected cells of *D. trinervis* actinorhizal nodules rather than in infected and meristematic cells (Figures 7B–E). Exploration of the model parameter space did not reveal a parameter configuration for which the biological auxin accumulation could be reproduced by the model based on DtAUX1 and DtPIN1 localization (data not shown). Altogether, our simulations indicate that a model based only on DtAUX1 and a DtPIN1-like activity is not able to predict the observed auxin accumulation and perception in *D. trinervis* nodules. Other transporters or mechanisms are therefore still missing.

## Discussion

Intercellular infection which is found in about 75% of actinorhizal genera and is regarded as an ancestral process which led to the more sophisticated root hair infection (Wall, 2000; Sprent, 2007; Madsen et al., 2010; Svistoonoff et al., 2014). Yet, very little is known about the mechanisms controlling intercellular infection in actinorhizal symbioses. As auxin has been involved in the intracellular infection of the actinorhizal plant *C. glauca*, here we analyzed the role of this phytohormone in the intercellularly infected *D. trinervis*. Our results indicate that auxin influx is important for nodule development during the symbiotic interaction between *D. trinervis* root and the soil actinomycete *Frankia* since treatment with the inhibitor of auxin influx 1-NOA lead to impaired and delayed nodule development compared to non-treated plants. This is similar to what has been described in *C. glauca* (Péret et al., 2007).

In order to analyze auxin involvement and auxin fluxes in more detail we looked for auxin in nodules using anti PAA antibodies. Previous studies denote the presence of auxins in nodules of root hair infected actinorhizal plants (Hammad et al., 2003; Perrine-Walker et al., 2010), mainly as phenylacetic acid (PAA) and indole-3-acetic acid (IAA). It has been reported that numerous strains of *Frankia* have the capacity to produce auxins (Wheeler et al., 1984; Berry et al., 1989; Hammad et al., 2003; Perrine-Walker et al., 2010). Interestingly, *Frankia* BCU110501, the symbiont of *D. trinervis* used as inoculum in our studies, produces auxins *in vitro* (Solans et al., 2011). In this work, we showed that the antibody against PAA labels *D. trinervis* nodule cells like in *C. glauca*, suggesting an accumulation of PAA related to infection of cells by *Frankia*. Nevertheless, we were unable to detect activation of auxin response in those cells using DR5. Thus, auxin in infected cells might be in the extracellular compartment, around *Frankia*, or inactive or DR5 is not functioning in the infected cells context. On the other hand, we found DR5 activation in the nodule meristem. It is remarkable that DR5 activation pattern in the lateral root or nodule meristems were not completely similar. While the expression appeared to be more continuous within the meristem of lateral root cell tissues, it showed a scattered pattern in meristem region of nodule primordia or mature nodules, suggesting again that although being related to lateral roots, actinorhizal nodules are novel and distinct root organs. Altogether DR5 activation takes place in the same cells as ProDtAUX1 either in lateral roots or nodules at different developmental stages.

*DtAUX1*, the putative orthologue of *CgAUX1*, showed a different activation pattern in *D. trinervis* nodules compared to *CgAUX1* in *C. glauca* (Péret et al., 2007, 2008). Whereas CgAUX1 is expressed in the vascular bundle and the infected cells in *C. glauca* nodules, ProDtAUX1 activation was restricted to the meristematic region in *D. trinervis* nodules. Remarkably, the activation of DtAUX1 in primary and lateral roots was identical to the one observed in *C. glauca* for *CgAUX1* and in Arabidopsis for *AtAUX1* (Marchant et al., 2002). We were unable to detect any GUS expression in *Frankia*-infected cells at early stages of infection or in nodules. Remarkably the same activation pattern was observed in roots and nodules expressing the *ProCgAUX1:GUS* fusion. These results suggest that auxin would not be involved in *D. trinervis* intercellular infection pathway where no infection thread formation is observed (Valverde and Wall, 1999), nor even at the stage of intracellular infection stage in mature symbiotic nodule tissue. This pattern of expression is substantially different to what happens in *C. glauca*, in which *CgAUX1* expression is closely related to the infection process, in root hairs and cortical cells infected by *Frankia* (Péret et al., 2007).

An alternative hypothesis could be that a paralogue of *DtAUX1* is expressed in infected cells. Although we tried to amplify *AUX* genes from nodule cDNA and used less stringent PCR conditions, we were unable to amplify any other gene than *DtAUX1*. Thus, only one gene belonging to the AUX-LAX family was identified in *D. trinervis*, whereas for *C. glauca* two genes have been reported (Péret et al., 2007). In *Datisca glomerata*, an actinorhizal plant belonging to the Cucurbitales order, two putative genes with high similarity to *AtAUX1* and *AtLAX2* were also identified (Demina et al., 2013). RNAseq developments will soon provide information about the existence of more genes similar to *DtAUX1*. Another difference in the auxin transporters distribution between the intercellular infected *D. trinervis* and the intracellular infected *C. glauca* appeared in the expression of PIN1 related proteins. The distribution of PIN1 in membranes of infected cells in *D. trinervis* contrasted with its expression in non-infected surrounding cells in *C. glauca* nodules (Perrine-Walker et al., 2010).

As stated before, PAA was detected in infected cells in *D. trinervis* nodules. Phenyl-acetate hopanetetrol is a hopanoid lipid that is present specifically in the envelope of *Frankia* vesicles (Berry et al., 1993), which are specialized multicellular structures that functions as protection barrier for enzyme nitrogenase against oxygen diffusion. Vesicles preferentially develop in nodules and they are associated to nitrogen fixation. PAA is required for the synthesis of hopanoid lipids, and its mobilization to the newly infected cortical cells may enable differentiation of *Frankia* hyphae into vesicles. PIN1 could be implicated in the transport of PAA from the mature infected cells, were *Frankia* is already differentiated into vesicles, to the surrounding cortical cells. Among these, PAA may facilitate the infection process by driving cell growth and cell wall remodeling (Perrine-Walker et al., 2010), and promote bacteria differentiation into vesicles.

Altogether, our results suggest that auxin transport in *D. trinervis* nodule differs from the one described in *C. glauca*. This suggests that the role of auxin is different in intracellular and intercellular infection in actinorhizal symbioses.

### Conflict of interest statement

The authors declare that the research was conducted in the absence of any commercial or financial relationships that could be construed as a potential conflict of interest.
