# Supplementary material for: Role of auxin during intercellular infection of Discaria trinervis by Frankia
Source: Front Plant Sci. 2014 Aug 21;5:399. doi: 10.3389/fpls.2014.00399 (PMC4139986; doi:10.3389/fpls.2014.00399)
Supplement: Supplementary file 1 [file Presentation1.PDF]

Supplementary Material

Role of auxin during intercellular infection of *Discaria trinervis* by Frankia

Imanishi L.[1,2], Perrine-Walker F.[2], Ndour A.[3], Vayssieres A.[2], Conejero G.[4], Lucas M.[2], Champion A.[2,3], Laplaze L.[2,3], Wall L.[1], Svistoonoff S.[2,3]*

[1]Laboratorio de Bioquímica, Microbiología e Interacciones Biológicas en el Suelo (LBMIBS), Universidad Nacional de Quilmes, Bernal, Argentina
[2]Groupe Rhizogenèse, UMR DIADE, Montpellier, France
[3]LAPSE, Dakar, Senegal
[4]Plateforme PHIV, Cirad, Montpellier, France

*** Correspondence:** Sergio Svistoonoff, Groupe Rhizogenèse, UMR DIADE, Institut de Recherche pour le Développement, (IRD), 911 Av. Agropolis 34394 Montpellier, France.
sergio.svistoonoff@ird.fr

Supplementary Figures

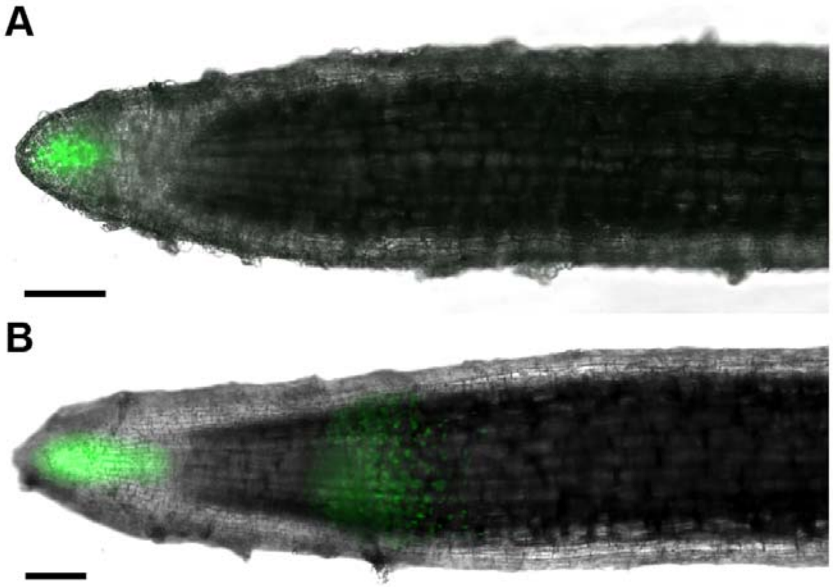

Supplementary figure 1. *DR5:VENUS:NLS* expression in *D. trinervis* roots after incubation with 10 µM NAA. Pictures were taken 24h after the addition of auxin. (A) Control roots with endogenous auxin, **(B)** treated roots. Scale bar: 100 µm.

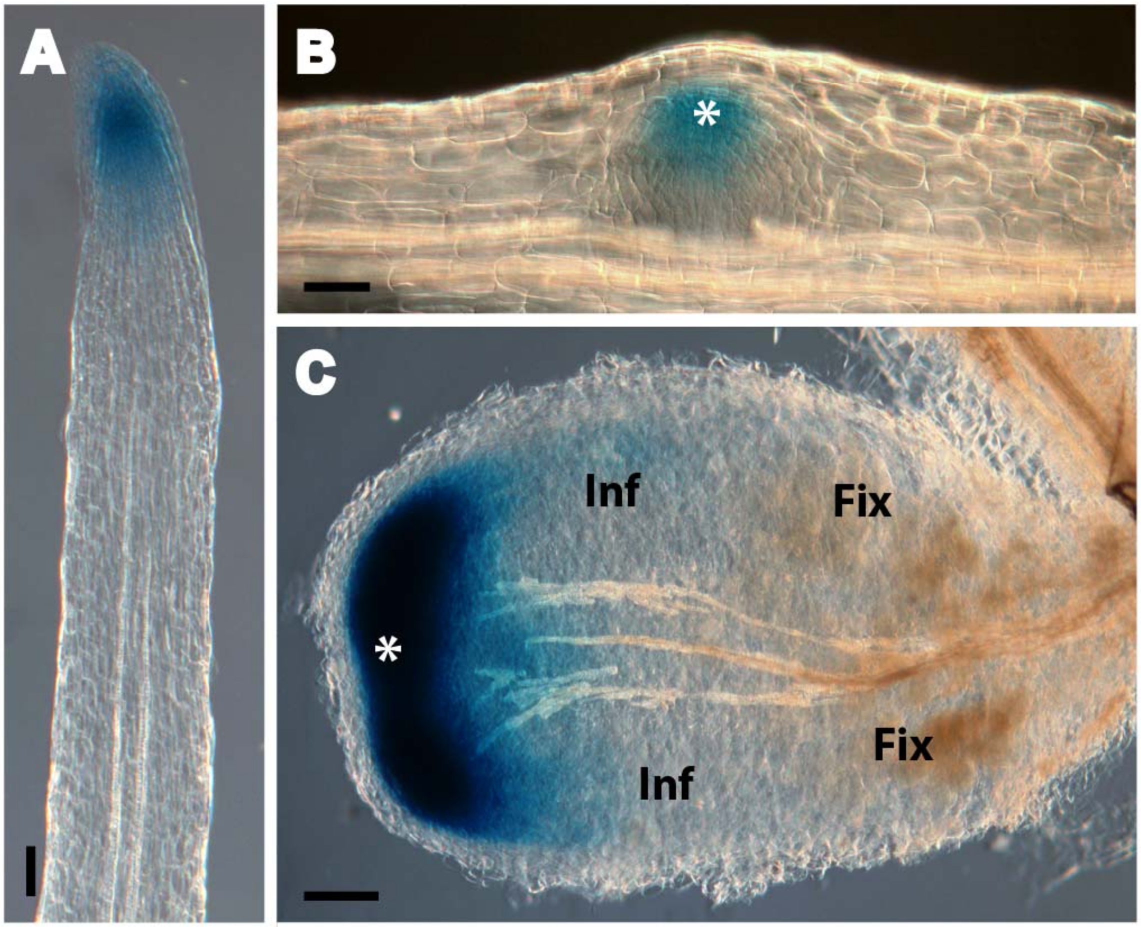

Supplementary figure 2. ***Pro$_{CgAUX1}$*:*GUS* expression in *D. trinervis*.** **(A)** Non-inoculated lateral root showing blue staining in the root tip. **(B)** Nodule primordium 5 dai. GUS activity is detected in the meristematic cells (asterisk). **(C)** Longitudinal section of a mature nodule 21 dai. ProCgAUX1 activity is observed in the meristematic region (asterisk) and decreases gradually through the infection zone (Inf) and is not detected in the fixation zone (Fix) where hypertrophied cells containing *Frankia* are localized. Scale bar: 100 µm.
